# Reliability and Variability of tDCS Induced Changes in the Lower Limb Motor Cortex

**DOI:** 10.3390/brainsci6030026

**Published:** 2016-07-27

**Authors:** Sangeetha Madhavan, Aishwarya Sriraman, Sally Freels

**Affiliations:** 1 Department of Physical Therapy, University of Illinois at Chicago, 1919 W. Taylor St., Chicago, IL 60612, USA; 2 Department of Physical Therapy, University of Illinois at Chicago, 1919 W. Taylor St., MC 898, Chicago, IL 60612, USA; aishsriraman@gmail.com; 3 Department of Epidemiology and Biostatistics, University of Illinois at Chicago, 1603 W. Taylor Street, Chicago, IL 60612, USA; sallyf@uic.edu

**Keywords:** TMS, tDCS, lower limb, motor cortex, tibialis anterior, reliability, variability

## Abstract

Background: Transcranial direct current stimulation (tDCS) is emerging as a promising adjuvant to enhance motor function. However, there has been increasing reservations about the reliability and variability of the neuromodulatory effects evoked by tDCS. Objective/Hypothesis: The main purpose of this study was to explore the test-retest reliability and inter-individual variability of tDCS of the lower limb M1 and the relationship between transcranial magnetic stimulation (TMS)-related measures and tDCS-induced changes. Methods: Fifteen healthy participants received anodal tDCS of the lower limb M1 either when performing a lower limb motor task or when the limb was at rest. Each condition was tested twice. tDCS induced changes in corticomotor excitability of the tibialis anterior muscle were measured using TMS. A repeated measures ANOVA was performed to examine efficacy of tDCS between the two task conditions. Intraclass correlation coefficients (ICC) and variance component analyses were performed to examine reliability and variability respectively. Results: A significant increase in in corticomotor excitability was noted for the tDCS-task condition at 140% active motor threshold (AMT) and when comparing recruitment curve slopes, but not at 120% and 130% AMT. Overall, ICC values between testing days for each stimulation condition ranged from 0.6–0.9. Higher ICCs were seen for higher TMS intensities (140% AMT) and recruitment curve slopes. Inter-individual variability contributed to 34% of the exhibited variance. Conclusions: Our data suggest that the TMS-related measure used to assess neuromodulation after tDCS has an effect on its perceived test-retest reliability and inter-individual variability. Importantly, we noticed that a high reliability and low variability does not necessarily indicate clinical efficacy of tDCS as some participants showed little to no modulation of corticomotor excitability consistently.

## 1. Introduction

Transcranial direct current stimulation (tDCS) is a simple and safe non-invasive brain stimulation technique that has been demonstrated to modulate neuronal excitability of the motor cortex (M1) with associated improvements in motor learning and motor function in individuals with and without neurological impairments [1,2]. Over the last decade, numerous studies have examined the effects of tDCS of the motor cortex on motor training and performance [3,4,5,6], explored optimization of tDCS parameters, such as electrode size, current density, and intensity [7,8], and examined its effects on functional improvements in neurological populations [9,10]. While all these studies have provided important information about the behavioral effects of tDCS, there still exists a big gap in knowledge regarding the robustness and consistency of the neurophysiological effects of tDCS. 

Transcranial magnetic stimulation (TMS) is typically used to measure the neurophysiological after-effects of tDCS on the motor cortex. Horvath et al. (2015), in a recent review about the reliability of tDCS on TMS measures in healthy adults, noted that the consistency of tDCS induced changes was poor for most TMS measures [11]. This observation is further supported by other studies that have examined the inter- and intra-individual variability of tDCS in healthy participants, and report considerable inter-individual variation with only 45%–50% of individuals demonstrating the expected up-regulation of motor evoked potential (MEP) amplitude in response to facilitatory tDCS of the upper limb M1 [12,13,14,15]. In these previous studies, tDCS was applied to the upper limb M1 when the targeted muscle was at rest. In the present study, we report the reliability and variability of tDCS on lower limb corticomotor plasticity and also examine the effectiveness of tDCS under different task conditions. The current study was motivated by our long-term interest in the development of noninvasive brain stimulation as an adjuvant to walking therapy after a stroke. Given the anatomical differences between the upper and lower M1 (i.e., variations in depth and density of cortical neurons, and projections of descending pathways) [16], it is important to specifically understand the reliability of tDCS-induced neuromodulation of the lower limb M1. We recently showed that timing of stimulation is a critical factor influencing the after effects of tDCS. Lower limb motor performance (accuracy in performing a skilled visuomotor task) was considerably enhanced when tDCS was applied during motor practice than at rest [5]. Hence, in this study, we aimed to examine the test-retest reliability and inter-individual variability of lower limb M1 tDCS under two task conditions, i.e., motor activity and rest. The TMS parameter used to measure the after effects of tDCS is also a critical factor that determines the consistency of the effect [17]. Hence, in the present study, we also explored the relationship between TMS-related measures on the test-retest reliability and inter-individual variability of lower limb M1 tDCS after effects.

## 2. Experimental Section

### 2.1. Selection Criteria

Fifteen participants with no neurological disorders (5 males, 10 females, age range 22–32 years) were recruited to participate in this study. A description of the study was provided and written consent approved by the University of Illinois Institutional Review Board in accordance with the declaration of Helsinki was obtained from each participant. The inclusion criteria for the study included no neurological disorders, full range of motion of the testing ankle joint, and between 18–35 years of age. The exclusion criteria included contraindications to TMS such as metal implants, cardiac pacemakers, unexplained headaches, history of seizures or epilepsy, and medications likely to alter cortical excitability. Leg dominance was noted by asking participants which leg they preferred to use to kick a ball. Thirteen participants reported to be right dominant and two participants reported to be left dominant. 

### 2.2. Study Design

**Figure 1:**
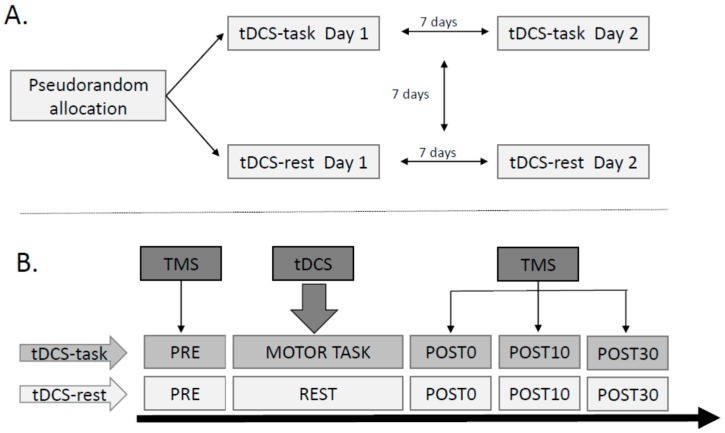
Schematic of the study design. (**A**) Illustrates the repeated measures study design; (**B**) illustrates the testing time points within each experimental session. Participants received anodal tDCS (1 mA for 15 min) while performing a motor task with their ankle or while at rest. Each stimulation condition was performed twice. Baseline and post-tDCS TMS measurements were performed.

This was a repeated-measures two factorial study design in which corticomotor excitability related variables were obtained twice (test-retest: Day 1 and 2) under two stimulation conditions (Figure 1). The two stimulation conditions were: (i) anodal tDCS during a skilled motor task (tDCS-task) and (ii) anodal tDCS at rest (tDCS-rest). Each participant was tested twice under each stimulation condition (total of 4 experimental sessions per participant). All testing sessions were separated by a minimum of seven days. All four stimulation conditions were pseudo-randomized between participants to avoid order effects.

During each session, the participant was seated comfortably in a chair with his/her non-dominant leg strapped to a custom built tracking device. Muscle activity was recorded from the non-dominant tibialis anterior (TA) muscle belly using surface electromyography. The non-dominant leg was chosen to avoid ceiling effects from the dominant side which maybe already be optimally active [18]. In the tDCS-task condition, tDCS was applied for 15 min while the participant practiced a visuomotor ankle tracking task using ankle dorsiflexion and plantar flexion. In the tDCS-rest condition, participants received tDCS for 15 min when he/she performed no activity with the leg. Corticomotor excitability before and after stimulation was examined using TMS. 

### 2.3. Instrumentation

#### 2.3.1. Electromyography (EMG)

Surface Ag/AgCl electrodes were placed over the muscle belly of the TA. The reference electrode was placed over the spinous process of the seventh cervical vertebrae. Before placing the EMG electrodes, the skin was shaved if needed, and rubbed with alcohol to reduce skin impedance. All EMG data were sampled at 2000 Hz, amplified 1000× and band pass filtered (10–500 Hz) with a Delsys EMG system (Bagnoli 8, Delsys, INC., Natick, MA, USA). Data were recorded using Spike2 software (Cambridge Electronic Design, Cambridge, UK). 

#### 2.3.2. Transcranial Direct Current Stimulation (tDCS)

A simple form of constant current stimulator (Chatanoonga Ionto, DJO Global, Guildford Surrey, UK) was used to deliver 1 mA of direct current for 15 min. Before the placement of electrodes the area was cleaned with alcohol. A 5 cm × 2.5 cm oblong saline soaked sponge electrode was placed over the leg area of M1, identified by hot spotting for the left (non-dominant) tibialis anterior muscle using single pulse TMS. A 7 cm × 5 cm carbonized electrode was placed over the dominant supraorbital region. 

#### 2.3.3. Transcranial Magnetic Stimulation (TMS)

TMS was applied using a single-pulse stimulator (Magstim, Dyfed, Wales, UK) via a 110 cm double cone coil using a posterior-anterior current orientation. Spike2 software was used to trigger the stimulator and also record the trigger pulses. During TMS, participants were provided with visual feedback of their muscle activity and instructed to maintain a tonic contraction of the non-dominant TA that represented 20% maximum voluntary contraction (MVC). MVC trials were performed at the beginning of each session. During the MVC trials, participants were instructed to pull as hard as possible with their foot (dorsiflexion) against resistance and the best EMG activity of 3 MVCs was noted. The double-cone coil was placed on the participant’s head, with the intersection of the two embedded coils on the vertex (intersection of the lines connecting the nasion and inion and the bilateral pretragus). With the participant contracting at 20% MVC, the coil was moved in small (~0.2 cm) increments to the position where the active motor threshold (AMT) was lowest for the TA. AMT was defined as the minimum stimulus intensity resulting in identifiable MEPs of at least 0.4 mV peak to peak in 50% of successive trials from the contralateral TA [5,19,20]. This position was marked as the “hotspot” for the TA. The active tDCS electrode was then positioned over the hotspot. A tight fitting cap was placed on the participant’s head above the tDCS electrodes. The hotspot was reconfirmed using TMS and active motor threshold was recalculated over the saline sponge active electrode. Pre and post TMS measurements were performed with the TMS coil over the electrode. Corticomotor excitability of the non-dominant TA muscle representation was assessed using single pulse TMS at a frequency of 0.25 Hz at 80%, 90%, 100%, 110%, 120%, 130% and 140% AMT for each participant. Eight MEPs were recorded at each intensity prior to tDCS stimulation (PRE), immediately post stimulation (POST0), ten minutes after the end of stimulation (POST10), and 30 min after the end of stimulation (POST30). Approximately 10 s of rest was provided between each intensity block. Blocks of intensities were collected in a random order.

To maintain consistent electrode and coil positioning across sessions, detailed distance recordings of the hotspot were made from the nasion, inion and bilateral pre-tragus to the vertex with and without the cap on. For all four testing sessions, TMS-related measures were obtained from the same spot to minimize variability in effects observed due to different testing positions for each day [21,22]. This position was also confirmed with TMS functionally at the beginning of the subsequent sessions and there was no necessity to change the hotspot for any of the participants. The tDCS electrode was also placed on the same spot for the above mentioned reason.

#### 2.3.4. Ankle Motor Task

We used a custom built manipulandum for ankle motor practice [5,19]. This device consisted of two adjustable plates and straps to secure the foot and shank in place. Participants performed ankle dorsiflexion and plantarflexion to match a sinusoidal wave on the computer screen as accurately as possible. A computer generated a waveform at a random frequency (0.2–0.4 Hz) and amplitude (60%–80% of the individual’s maximum comfortable range of motion (ROM)). The participant’s ROM was measured at the beginning of each session using the potentiometer built in the manipulandum. For the tDCS-task condition, the participants performed the motor task with a random waveform sequence for 15 min with a one-minute rest interval after every four minutes. For the tDCS-rest condition, participants were instructed to maintain the foot at rest and relax. 

### 2.4. Data Analyses

All data were imported and analyzed by Spike2 software. The following TMS-related measures were evaluated: (i) peak to peak MEP amplitude at each intensity; (ii) MEP area at each intensity; and (iii) linear slope of recruitment curve (RC), for MEP amplitude and MEP area. A MEP window was established for each participant, for the pre and post TMS trials during each session, by finding the onset and offset latencies of a large MEP in response to the highest TMS intensity (140% AMT). The same MEP window was then applied to analyze all the MEPs within a given session. MEP amplitude was calculated as the peak to peak magnitude of EMG activity within the MEP window. MEP area was calculated as the rectified integrated area of the EMG within the time window. Both MEP area and amplitude were then averaged across the eight MEPs for each TMS intensity, each time point, and each participant. The average MEP response was plotted against the corresponding stimulus intensity, and a linear function was used to fit this recruitment curve. Since we chose not to stimulate at intensities that elicit maximum MEPs, we used a conservative linear fit rather than a Boltzman fit, accepting the likelihood of not detecting a difference in slope. The linear slope of this recruitment curve was calculated.

### 2.5. Statistical Analyses

All statistical analyses were performed using Statistical Package for the Social Sciences (SPSS, IBM software version 22, Armonk, NY, USA). Statistical significance was considered at *p* < 0.05. The following analyses were performed.

#### 2.5.1. Effects of Anodal tDCS

We compared change in MEP values (area and amplitude) before and after tDCS between tDCS-task and tDCS-rest conditions using a 2 × 2 × 3 mixed model ANOVA with factors: condition (tDCS-task vs. tDCS-rest), day (Day 1 and 2) and time (POST0, POST10 and POST30). The change scores were nested within the day factor. Tukey’s post hoc analyses were conducted if ANOVA revealed a significant main effect or interaction between factors.

#### 2.5.2. Reliability of Anodal tDCS-Induced Changes

Interclass Correlation Coefficients (ICC) were used to estimate the test-retest reliability for Day 1 vs. Day 2 within each stimulation condition. ICCs were run using a two-way random analysis for absolute agreement. ICC is the ratio of between-subject variation to the total variation, and it is the statistical measure most adopted for quantifying test-retest reliability [23]. ICC scores ranges from 0–1 with a value of 1 indicating perfect reliability, ≥0.90 considered to be excellent, 0.75–0.89 to be good, ≤0.74 to be moderate, and below 0.5 to be poor. ICC comparisons were made for MEP amplitude and MEP area measures at selected individual intensities (120%, 130% and 140% AMT), and for linear slopes of MEP amplitude and MEP area RC curves. 

We also categorized the MEP amplitude data into responders (R) and non-responders (NR) to tDCS for the TIME bins and TMS measures that revealed significant modulation in the main model. We classified subjects as R or NR based on the grand average normalized MEP (average of all MEPs at the post time points divided by pre) greater or lesser than 1, respectively [12,13,14,15].

#### 2.5.3. Intra-and Inter-Individual Variability

We used a variance component analyses (ANOVA type) to determine intra-and inter-individual variability of TMS-related measures. Normalized MEP amplitude was the dependent factor, “day” and “subject” were considered random factors and “time” was a fixed factor. We adopted this analysis as it has been previously used by studies investigating reliability of non-invasive brain stimulation measures [14,24].

## 3. Results

All participants tolerated the experiment well. Apart from the mild tingling sensation perceived during ramping of the current, participants reported no adverse effects. 

### 3.1. Effects of Anodal tDCS

**Figure 2:**
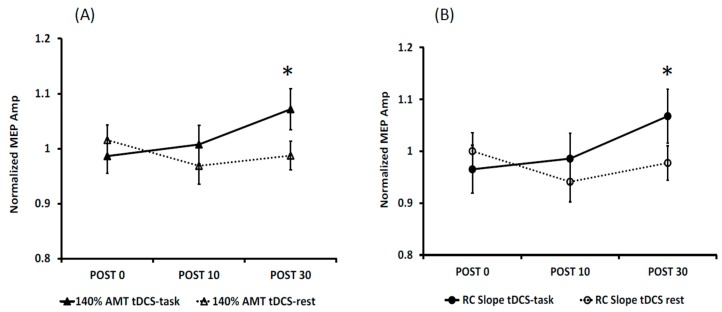
MEP amplitudes normalized to baseline values at 140% AMT (**A**) and RC slopes (**B**) for the tDCS-task and tDCS-rest conditions. An average of Day 1 and 2 values are represented to show the significant interaction of Condition and Time. * denotes a significant difference between the two conditions at POST30 (*p* < 0.05).

MEP amplitude: The 3-way ANOVA revealed no significant interaction or main effects for 120% AMT and 130% AMT. The interaction effect of condition and time was significant for 140% AMT (F(1, 145) = 4.3, *p* = 0.039) and for RC slopes (F(1, 145) = 3.9, *p* = 0.05) (Figure 2). For both 140% AMT and RC slope, post hoc analyses revealed a significantly higher mean (9% increase) for tDCS-task compared to tDCS-rest at the POST30 time point. 

**Figure 3:**
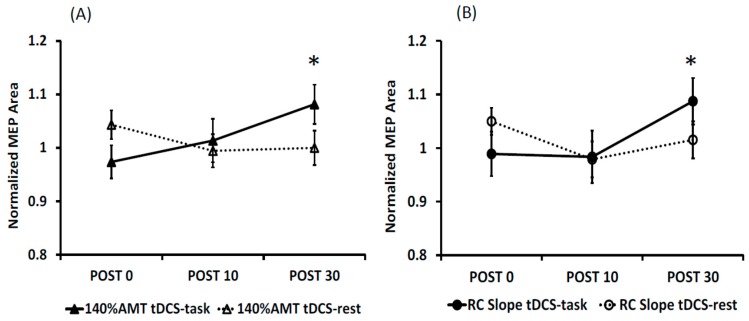
MEP areas normalized to baseline values at 140% AMT (**A**) and RC slopes (**B**) for the tDCS-task and tDCS-rest conditions. An average of Day 1 and 2 values are represented to show the significant interaction of Condition and Time. * denotes a significant difference between the two conditions at POST30 (*p* < 0.05).

MEP area: The MEP area data showed a similar trend as MEP amplitude (Figure 3). The 3-way ANOVA revealed no significant interaction or main effects for 120% AMT and 130% AMT. The interaction effect of stimulation and time was significant at 140% AMT (F(1, 145) = 5.06, *p* = 0.019) and for RC slopes (F(1, 145) = 4.4, *p* = 0.045) (Figure 3). For 140% AMT, post hoc analyses revealed a significantly higher mean for tDCS task (9%) compared to tDCS-rest at POST30. Similarly, for RC slope, a significant higher mean was noted for tDCS-task (7%) at POST30 than tDCS-rest condition.

### 3.2. Reliability of tDCS-Induced Changes

**Figure 4:**
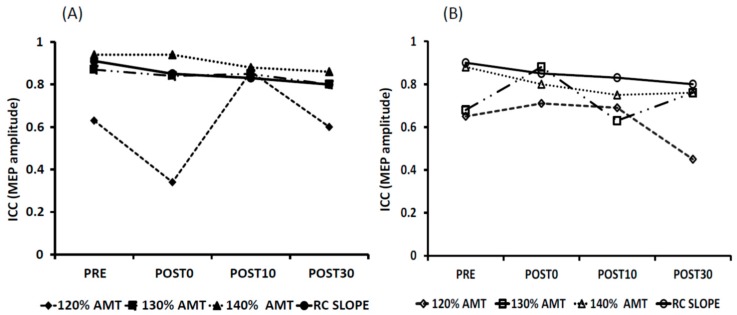
Graphical representation of ICC values for MEP amplitudes at 120%, 130%, 140% AMT and RC slope for all the time points for tDCS-task (**A**) and tDCS-rest (**B**). Note the decreased ICC values at 120% AMT.

MEP amplitude: The ICC values for MEP amplitudes are shown in Table 1 and in Figure 4. Overall, both stimulation conditions showed good to excellent ICC values for Day 1 vs. Day 2 at 140% AMT and for the RC slope. The ICC values were found to be poor to moderate for the 120% and 130% AMT. All ICC values (except for tDCS-task at POST0 and tDCS-rest at POST30 at 120% AMT) were statistically significant.

**Figure 5:**
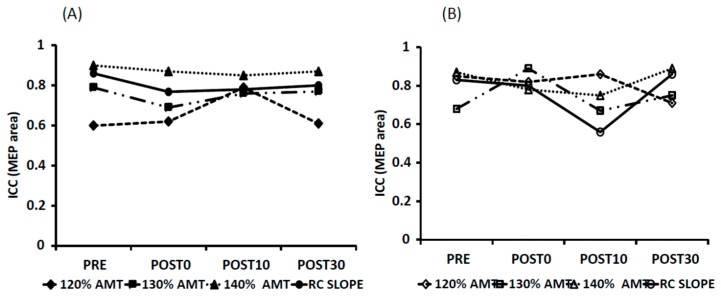
Graphical representation of ICC values for MEP areas at 120%, 130%, 140% AMT and RC slope for all the time points for tDCS-task (**A**) and tDCS-rest (**B**). Note the decreased ICC values at 120% AMT.

MEP Area: The ICC values for MEP area are shown in Table 2 and Figure 5. Similar to MEP amplitude, both stimulation conditions showed overall good to excellent ICC values for Day 1 vs. Day 2 at 140% AMT and for the RC slope. The ICC values were found to be poor to moderate for the 120% and 130% AMT. All the ICC values were statistically significant. 

Responders vs. Non-responders: The number of responders and non-responders is reported in Table 3. Overall (average of Day 1 and 2), the number of responders were greater for the tDCS-task condition (66% RC slope; 60% 140% AMT) compared to tDCS-rest (43% RC slope; 47% 140% AMT). Approximately 47% responded with consistent facilitation between both task conditions.

### 3.3. Intra- and Inter-Individual Variability

**Figure 6:**
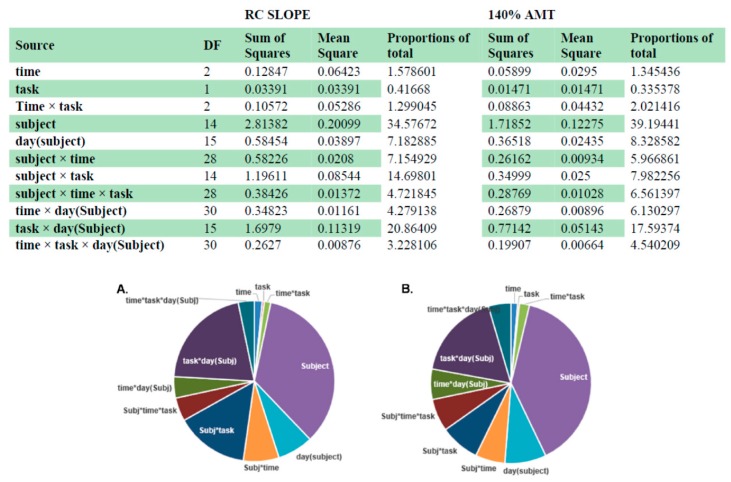
Contribution of inter- and intra-individual variability to the total variance for MEP amplitude for RC slope and 140% AMT. The table represents the degrees of freedom (DF), sum of squares, mean squares and proportions of total for each factor used in the analyses. The pie chart illustrates the proportions of total. i.e., contribution of sum of squares to the total sum of squares for RC slope (**A**) and 140% AMT (**B**).

The results of the variance component analyses of the MEP amplitudes at 140% AMT and RC slope measurements for Post30 are shown in Figure 6. Inter-individual variability (“subject” factor) had the greatest contribution to variance (~36.5%) compared to other factors including intra-individual variability (“day (subject)” factor).

## 4. Discussion

This is the first study to examine test-retest reliability and inter-individual variability of tDCS induced corticomotor excitability changes in the healthy lower limb M1 during motor activity (tDCS-task) versus the absence of motor activity (tDCS-rest). Our results suggest that overall tDCS has fair test-retest reliability when applied with or without a motor task (as observed by the high ICC scores). This reliability is higher when tDCS induced changes are measured at higher TMS intensities (140% AMT) or when considering recruitment curve slopes. Both MEP amplitude and area measurements show similar trends in test-retest reliability. The changes in corticomotor excitability between the two stimulation conditions (tDCS-task vs. tDCS-rest) were most apparent at 140% MSO and with RC slopes 30 min after stimulation. The tDCS-task condition showed greater upregulation than tDCS-rest. However, we noted that the amount of upregulation induced by tDCS was overall minimal (~9%), suggesting that the high reliability results shown here may not be meaningful when considering group level analyses. The variance component analyses revealed that the inter-individual variations was the greatest contributor to overall variability.

### 4.1. Reliability of TMS as an Experimental Tool

Before we can elaborate on the consistency of tDCS after effects, it is important that we evaluate the robustness of TMS as a tool utilized in measuring the neurophysiological effects of tDCS. Previous studies have shown TMS to be reliable for evaluating corticomotor excitability for both upper limb and lower limb muscles [25,26,27,28]. The test-retest reliability of MEPs of the tibialis anterior muscle has been observed to be between 0.7–0.8 in the healthy population [28,29]. Similarly in the present study, we observed moderate to high ICC values (0.6–0.9) for the TA muscle at baseline indicating a high reliability of TMS as a tool for examining corticomotor excitability of the TA muscle. This strong reliability of the baseline data suggests that any changes noted in the post measurements can be attributed to changes evoked by tDCS or inherent biological variability of the individual, and limited to the measurement variability of TMS. 

Alonzo et al. (2012) reported variable TMS-related baseline measures when tDCS was applied on consecutive days compared to less variable baseline values when stimulation was applied after a delay of 48 h [30]. Galvez et al. (2013) reported higher variability in TMS-related baseline measures during consecutive sessions of tDCS [31]. The increased variability in TMS-related baseline values for consecutive sessions of tDCS has been explained to be due to cumulative increase in cortical excitability that could have been preserved between stimulation sessions. The seven day minimum period between our test-retest sessions helped eliminate the possibility of retention of these cumulative effects associated with consecutive stimulation. This may also explain the high consistency we report for the tDCS-induced modulations.

### 4.2. Reliability of tDCS

To understand the reliability of the after effects of tDCS, comparisons between the two testing days within the same stimulation condition (task vs. rest) was performed. The ICC comparisons at 120% AMT, 130% AMT, 140% AMT and RC slopes showed moderate to excellent reliability for both task conditions at all the time points. This strong test-retest reliability of tDCS-induced modulations, suggests that the amount of change demonstrated by each individual was consistent between the two days. An interesting finding was that the ICC values increased with increasing stimulus intensity with moderate reliability at 120% AMT and 130% AMT, good reliability for the RC slope, and excellent reliability at 140% AMT for both the stimulation conditions. A reason for decreased reliability at the lower stimulus intensities is possibly due to the larger trial to trial variability associated with the TMS-related outcome measures at these intensities. The inherent folding of the cerebral cortex means that only certain neurons are stimulated at low intensities resulting in lower representation of neurons modulated by tDCS. As the stimulation intensity increases, it is possible that there is a greater representation of neurons that are upregulated by tDCS resulting in higher reliability. The test-retest reliability in the current study is considerably better than other upper extremity tDCS reliability studies, such as Lopez-Alonzo et al. (2015) [14] and Chew et al. (2015) [15]. Chew et al. (2015) noted a lack of test-retest reliability when stimulation was performed at 0.5 mA at rest [15]. In their study, a TMS parameter of 130% resting MT was used when the muscle was at rest. Lopez-Alonso (2015) [14] reported none-fair reliability while they stimulated and tested a muscle at rest with a TMS intensity that evoked a 1 mV MEP [14]. The higher reliability in our study could be attributed not only to a different area of motor cortex that was stimulated but also because we used different TMS intensities for testing the active muscle. Approximately 47% of our participants responded with the expected upregulation to tDCS under both task conditions. This is in line with other studies that have reported similar percentages of responders [12,13]. 

### 4.3. Variability of tDCS

We found that inter-individual variance was the largest contributor to the total variability of tDCS-induced neuromodulation. This is also been demonstrated by previous studies [14,15,24]. It is likely that this considerable inter-individual variability is due to a variety of factors some of which include individual differences in neuro anatomy and physiology, genetic factors [32], hormonal influences [33] and gender [34,35]. The amount of intra-individual variability (day (subject) was relatively lower (~7%)). In the clinical context, it is important to keep in mind that not everybody is expected to show desired upregulation to tDCS and an initial session to determine if a person is responsive maybe necessary. In addition, future work should examine individualization of tDCS electrode montage and dosage to improve efficiency of neuromodulation.

### 4.4. Comparison of Corticomotor Changes between the Two Task Conditions

In a previous study, we reported that when tDCS is applied in conjunction with a motor task, it improves motor performance better than when compared to tDCS applied prior to the motor task [5]. However, we did not observe any significant changes in corticomotor excitability. This has been typical for other tDCS studies where behavior is measurably enhanced compared to corticomotor excitability [19,36,37,38]. In most of these studies, including our previous studies, the preferred TMS-related measure used to quantify excitability has been 120% AMT. Correspondingly, in the present study, we did not observe any differences between the tDCS-task and tDCS-rest conditions at 120% for the MEP measures. However, significant differences were noted at 140% AMT and RC slope at the POST 30 time point, capturing changes in neuromodulation induced by the tDCS-task condition. A curious result was the lack of significant differences elicited by tDCS at POST0 and POST10. Previous studies, including studies from our laboratory, have shown that the facilitatory effects of anodal tDCS are seen immediately or within 10 min following cessation of stimulation which continues through later time points [5,12,13,19,38]. Jayaram and Stinear (2009) [39] noted that there is considerable between-subject variability in the time course of stimulation, with different individuals showing peak facilitation at different time points. It is possible that this inter individual variability in time course of modulation partly explains our results in the present study.

### 4.5. Is tDCS a Clinically Effective Tool?

In order for tDCS to be clinically effective, it should not only demonstrate high reliability and low inter-individual and intra-individual variability but the actual change induced should be sufficient to exceed the threshold to induce neural plasticity. This threshold maybe different for each individual and is currently an unquantified entity. In the present study, we observed that change from baseline for MEP amplitude and area ranged from approximately −17% to 25% for both stimulation conditions. We would like to highlight that although participants showed strong reliability of measures, i.e., consistent amount of change from one session to the next, the amount of change was minimal for some participants. For example, if an individual revealed an upregulation of 4% following tDCS, it can be expected that they would show a similar change of 4% in the following session. However, this amount of change may not be significant to cause any real alteration in the neuromotor system. It is also important to note that facilitatory tDCS induced down regulation in some participants. The absence of desired amounts of modulation in corticomotor excitability has been noted in other studies and is the subject of controversy in recent years questioning the effectiveness of tDCS [40]. Numerous studies have reported robust behavioral changes induced by tDCS with either non-significant changes in corticomotor excitability or absence of measurements with respect to corticomotor excitability [19,36,37,38,41]. In a recent review, Horvath et al. (2015) [11] reported low reliability of tDCS when pooling data from several different studies. Poor reliability of tDCS when comparing data across different studies can be explained by three factors: (1) stimulation related; (2) individual related and (3) outcome measures used. Stimulation-related factors include duration of stimulation, dosage of current, size of electrodes, timing of stimulation and task associated with stimulation. Individual factors include anatomical differences in cortical neurons, hair thickness, skull thickness, circadian rhythms, hormonal cycles, diurnal variations, and sweating. Outcome measures include the type of task used to test the effects of tDCS, TMS parameter used to measure excitability changes and the time (following the intervention) at which change was measured. In our study we controlled for some of these factors by testing the same individual with the same dosage and duration of stimulation under two different task conditions. We also controlled for outcome measures as a limiting factor by exploring neuromodulation with a variety of TMS-related measures. We tested corticomotor excitability during a tonic contraction (20% MVC) when variability of TMS has been suggested to be minimum [29,42]. However, we were unable to control for individual biological variations. Controlling for two out of three factors, ensured an increase in the reproducibility of the neuromodulatory effects of tDCS. However, the absolute amount of change in neuromodulation was wide-ranging suggesting further need for exploring optimization of stimulation effects. 

### 4.6. Limitations

A limitation of this study is the low sample size. However, our study design is strengthened by the repeated measure study design with 15 participants attending four sessions each. We acknowledge that this small sample size could limit the applicability of our results to a wider population. Another limitation is the lack of an appropriate sham/control condition. In the present study, we focused on examining the reliability of tDCS modulated by varying task conditions. We did not include a sham condition to reduce the burden on our participants. We also made this decision based on previous studies in our lab, which have reported the impact of tDCS in comparisons to sham conditions [5,19,20,43]. Nevertheless, this is an important limitation that needs to be addressed in the future. In addition, the results of our study are relevant only to the healthy lower limb M1 particularly the tibialis anterior muscle representation with a current dosage of 15 mA/min. We cannot extrapolate our findings to other tDCS configurations or other muscles. We chose to test this particular protocol because we have shown it be most effective in creating focal neuromodulation of the stimulated lower limb M1 [20,38,43]. We also cannot comment on the test-retest reproducibility of cathodal tDCS or in populations with a damaged nervous system. We report the results of a single session and cannot extrapolate these results to repetitive sessions as used in a clinical setting. Nevertheless, this is the beginning step to better understanding the neuromodulatory efficacy of tDCS of the lower limb M1.

## 5. Conclusions

In conclusion, the results of the present study provide a comparison between the reliability of tDCS induced corticomotor changes under two task conditions, tDCS-rest and tDCS-task. High test-retest reliability was observed for higher stimulus intensities (140% AMT) and recruitment curve slopes. We noticed considerable inter-individual variability in the responses. However, the absolute changes induced by tDCS was minimal, minimizing the significance of the reliability shown by tDCS. Future studies should consider evaluation of corticomotor excitability at higher TMS intensities or performing input-output response curves in order to obtain a better understanding of the neuromodulatory effects evoked with tDCS. It is important to keep in mind that although we showed high reliability of tDCS-induced modulations in corticomotor excitability at higher intensities, only 47% of individuals showed the desired up regulation under both task conditions. Additionally, the amount of upregulation was minimal for some individuals, which may not be sufficient to induce neural plasticity. Future studies will need to explore individual response patterns and develop more rigorous protocols to personalize tDCS parameters to each individual.

## Figures and Tables

**Table 1:** ICCs for MEP amplitudes.

Day 1 vs. Day 2	PRE		POST0		POST10		POST30	
	tDCS-Task	tDCS-Rest	tDCS-Task	tDCS-Rest	tDCS-Task	tDCS-Rest	tDCS-Task	tDCS-Rest
**120% AMT**	0.63 *	0.65 *	0.34	0.71 *	0.86 *	0.69 *	0.60 *	0.45
**130% AMT**	0.87 *	0.68 *	0.84 *	0.88 *	0.85 *	0.63 *	0.76 *	0.76 *
**140% AMT**	0.94 *	0.88 *	0.94 *	0.80 *	0.88 *	0.73 *	0.86 *	0.76 *
**RC SLOPE**	0.91 *	0.81 *	0.85 *	0.80 *	0.83 *	0.63 *	0.80 *	0.72 *

Presents ICCs for MEP amplitude at 120%, 130%, 140% AMT and RC slope between Day 1 and 2 for both the conditions. * denotes a significant *p* < 0.05.

**Table 2:** ICCs for MEP area.

Day 1 vs. Day 2	PRE		POST0		POST10		POST30	
	tDCS-Task	tDCS-Rest	tDCS-Task	tDCS-Rest	tDCS-Task	tDCS-Rest	tDCS-Task	tDCS-Rest
**120% AMT**	0.66 *	0.85 *	0.62 *	0.82 *	0.79 *	0.86 *	0.61	0.71 *
**130% AMT**	0.68 *	0.69 *	0.68 *	0.89 *	0.76 *	0.67 *	0.77 *	0.81 *
**140% AMT**	0.88 *	0.87 *	0.87 *	0.78 *	0.85 *	0.75 *	0.87 *	0.89 *
**RC SLOPE**	0.85 *	0.93 *	0.76 *	0.80 *	0.78 *	0.55	0.80 *	0.86 *

Presents ICCs for MEP area at 120%, 130%, 140% AMT and RC slope between Day 1 and 2 for both the conditions. * denotes a significant *p* < 0.05.

**Table 3:** Responders (R) and Non-responders (NR) in the Post30 time bin.

		tDCS-task		tDCS-rest	
		R	NR	R	NR
**140% AMT**	Day 1	8	7	8	7
	Day 2	10	5	6	9
	Facilitation for both task conditions	7			
**RC SLOPE**	Day 1	11	4	6	9
	Day 2	9	6	7	8
	Facilitation for both task conditions	7			

## References

[1] Nitsche M.A., Cohen L.G., Wassermann E.M., Priori A., Lang N., Antal A., Paulus W., Hummel F., Boggio P.S., Fregni F. (2008). Transcranial direct current stimulation: State of the art 2008. Brain Stimul..

[2] Madhavan S., Shah B. (2012). Enhancing motor skill learning with transcranial direct current stimulation—A concise review with applications to stroke. Front. Psychiatry.

[3] Nitsche M.A., Schauenburg A., Lang N., Liebetanz D., Exner C., Paulus W., Tergau F. (2003). Facilitation of implicit motor learning by weak transcranial direct current stimulation of the primary motor cortex in the human. J. Cogn. Neurosci..

[4] Reis J., Schambra H.M., Cohen L.G., Buch E.R., Fritsch B., Zarahn E., Celnik P.A., Krakauer J.W. (2009). Noninvasive cortical stimulation enhances motor skill acquisition over multiple days through an effect on consolidation. Proc. Natl. Acad. Sci. USA.

[5] Sriraman A., Oishi T., Madhavan S. (2014). Timing-dependent priming effects of tDCS on ankle motor skill learning. Brain Res..

[6] Stagg C.J., Jayaram G., Pastor D., Kincses Z.T., Matthews P.M., Johansen-Berg H. (2011). Polarity and timing-dependent effects of transcranial direct current stimulation in explicit motor learning. Neuropsychologia.

[7] Bastani A., Jaberzadeh S. (2013). a-tDCS differential modulation of corticospinal excitability: The effects of electrode size. Brain Stimul..

[8] Kidgell D.J., Daly R.M., Young K., Lum J., Tooley G., Jaberzadeh S., Zoghi M., Pearce A.J. (2013). Different current intensities of anodal transcranial direct current stimulation do not differentially modulate motor cortex plasticity. Neural Plast..

[9] Kuo M.F., Paulus W., Nitsche M.A. (2014). Therapeutic effects of non-invasive brain stimulation with direct currents (tDCS) in neuropsychiatric diseases. Neuroimage.

[10] Floel A. (2014). tDCS-enhanced motor and cognitive function in neurological diseases. Neuroimage.

[11] Horvath J.C., Forte J.D., Carter O. (2015). Evidence that transcranial direct current stimulation (tDCS) generates little-to-no reliable neurophysiologic effect beyond MEP amplitude modulation in healthy human subjects: A systematic review. Neuropsychologia.

[12] Lopez-Alonso V., Cheeran B., Río-Rodríguez D., Fernández-Del-Olmo M. (2014). Inter-individual variability in response to non-invasive brain stimulation paradigms. Brain Stimul..

[13] Wiethoff S., Hamada M., Rothwell J.C. (2014). Variability in response to transcranial direct current stimulation of the motor cortex. Brain Stimul..

[14] Lopez-Alonso V., Fernández-Del-Olmo M., Costantini A., Gonzalez-Henriquez J.J., Cheeran B. (2015). Intra-individual variability in the response to anodal transcranial direct current stimulation. Clin. Neurophysiol..

[15] Chew T., Ho K.A., Loo C.K. (2015). Inter- and intra-individual variability in response to transcranial direct current stimulation (tDCS) at varying current intensities. Brain Stimul..

[16] Lemon R.N. (2008). Descending pathways in motor control. Annu. Rev. Neurosci..

[17] Labruna L., Jamil A., Fresnoza S., Batsikadze G., Kuo M.F., Vanderschelden B., Ivry R.B., Nitsche M.A. (2016). Efficacy of anodal transcranial direct current stimulation is related to sensitivity to transcranial magnetic stimulation. Brain Stimul..

[18] Marquez J., Conley A., Karayanidis F., Lagopoulos J., Parsons M. (2015). Anodal direct current stimulation in the healthy aged: Effects determined by the hemisphere stimulated. Restor. Neurol. Neurosci..

[19] Shah B., Nguyen T.T., Madhavan S. (2013). Polarity independent effects of cerebellar tDCS on short term ankle visuomotor learning. Brain Stimul..

[20] Madhavan S., Stinear J.W. (2010). Focal and bidirectional modulation of lower limb motor cortex using anodal transcranial direct current stimulation. Brain Stimul..

[21] Herwig U., Padberg F., Unger J., Spitzer M., Schönfeldt-Lecuona C. (2001). Transcranial magnetic stimulation in therapy studies: Examination of the reliability of “standard” coil positioning by neuronavigation. Biol. Psychiatry.

[22] Sparing R., Buelte D., Meister I.G., Paus T., Fink G.R. (2008). Transcranial magnetic stimulation and the challenge of coil placement: A comparison of conventional and stereotaxic neuronavigational strategies. Hum. Brain Mapp..

[23] Bartko J.J. (1966). The intraclass correlation coefficient as a measure of reliability. Psychol. Rep..

[24] Sommer M., Wu T., Tergau F., Paulus W. (2002). Intra- and interindividual variability of motor responses to repetitive transcranial magnetic stimulation. Clin. Neurophysiol..

[25] Malcolm M.P., Triggs W.J., Light K.E., Shechtman O., Khandekar G., Gonzalez Rothi L.J. (2006). Reliability of motor cortex transcranial magnetic stimulation in four muscle representations. Clin. Neurophysiol..

[26] Christie A., Fling B., Crews R.T., Mulwitz L.A., Kamen G. (2007). Reliability of motor-evoked potentials in the ADM muscle of older adults. J. Neurosci. Methods.

[27] Carroll T.J., Riek S., Carson R.G. (2001). Reliability of the input-output properties of the cortico-spinal pathway obtained from transcranial magnetic and electrical stimulation. J. Neurosci. Methods.

[28] Van Hedel H.J., Murer C., Dietz V., Curt A. (2007). The amplitude of lower leg motor evoked potentials is a reliable measure when controlled for torque and motor task. J. Neurol..

[29] Cacchio A., Cimini N., Alosi P., Santilli V., Marrelli A. (2009). Reliability of transcranial magnetic stimulation-related measurements of tibialis anterior muscle in healthy subjects. Clin. Neurophysiol..

[30] Alonzo A., Brassil J., Taylor J.L., Martin D., Loo C.K. (2012). Daily transcranial direct current stimulation (tDCS) leads to greater increases in cortical excitability than second daily transcranial direct current stimulation. Brain Stimul..

[31] Galvez V., Alonzo A., Martin D., Loo C.K. (2013). Transcranial direct current stimulation treatment protocols: Should stimulus intensity be constant or incremental over multiple sessions?. Int. J. Neuropsychopharmacol..

[32] Antal A., Chaieb L., Moliadze V., Monte-Silva K., Poreisz C., Thirugnanasambandam N., Nitsche M.A., Shoukier M., Ludwig H., Paulus W. (2010). Brain-derived neurotrophic factor (BDNF) gene polymorphisms shape cortical plasticity in humans. Brain Stimul..

[33] Inghilleri M., Conte A., Currà A., Frasca V., Lorenzano C., Berardelli A. (2004). Ovarian hormones and cortical excitability. An rTMS study in humans. Clin. Neurophysiol..

[34] Li L.M., Uehara K., Hanakawa T. (2015). The contribution of interindividual factors to variability of response in transcranial direct current stimulation studies. Front. Cell. Neurosci..

[35] Ridding M.C., Ziemann U. (2010). Determinants of the induction of cortical plasticity by non-invasive brain stimulation in healthy subjects. J. Physiol..

[36] Tecchio F., Zappasodi F., Assenza G., Tombini M., Vollaro S., Barbati G., Rossini P.M. (2010). Anodal transcranial direct current stimulation enhances procedural consolidation. J. Neurophysiol..

[37] Gomes-Osman J., Field-Fote E.C. (2013). Bihemispheric anodal corticomotor stimulation using transcranial direct current stimulation improves bimanual typing task performance. J. Mot. Behav..

[38] Madhavan S., Weber K.A., Stinear J.W. (2011). Non-invasive brain stimulation enhances fine motor control of the hemiparetic ankle: Implications for rehabilitation. Exp. Brain Res..

[39] Jayaram G., Stinear J.W. (2009). The effects of transcranial stimulation on paretic lower limb motor excitability during walking. J. Clin. Neurophysiol..

[40] Horvath J.C., Carter O., Forte J.D. (2014). Transcranial direct current stimulation: Five important issues we aren’t discussing (but probably should be). Front. Syst. Neurosci..

[41] Vines B.W., Cerruti C., Schlaug G. (2008). Dual-hemisphere tDCS facilitates greater improvements for healthy subjects’ non-dominant hand compared to uni-hemisphere stimulation. BMC Neurosci..

[42] Darling W.G., Wolf S.L., Butler A.J. (2006). Variability of motor potentials evoked by transcranial magnetic stimulation depends on muscle activation. Exp. Brain Res..

[43] Devanathan D., Madhavan S. (2015). Effects of anodal tDCS of the lower limb M1 on ankle reaction time in young adults. Exp. Brain Res..

